# Ultra-Processed Foods and Chronic Kidney Disease: Is Inflammaging the Missing Link?

**DOI:** 10.3390/biom16050660

**Published:** 2026-04-29

**Authors:** Dimitris C. Kounatidis, Apostolos Evangelopoulos, Krystalia Dimitriou, Natalia G. Vallianou

**Affiliations:** 1 Diabetes Center, First Department of Propaedeutic Internal Medicine, Laiko General Hospital, Medical School, National and Kapodistrian University of Athens, 11527 Athens, Greece; dimitriskounatidis82@outlook.com; 2 Medical School, National and Kapodistrian University of Athens, 11527 Athens, Greece; apostolos.evangelopoulos.nak@gmail.com; 3 Second Department of Internal Medicine, NIMTS Hospital, 11521 Athens, Greece; k.dimitriou@gmial.com; 4 First Department of Internal Medicine, Sismanogleio General Hospital, 15126 Athens, Greece

**Keywords:** chronic kidney disease, gut dysbiosis, inflammaging, phosphate overload, policy measures, ultra-processed foods

## Abstract

Chronic kidney disease (CKD) is a progressive, irreversible condition that imposes a substantial burden of morbidity and mortality. While inadequate glycemic and blood pressure control remain its central drivers, dietary patterns are increasingly recognized as modifiable determinants of disease trajectory. Ultra-processed foods (UPFs), now pervasive in contemporary diets, have attracted particular attention due to their distinct physicochemical properties and biological effects. These products are industrial formulations that undergo multiple processing steps and are typically characterized by low nutritional quality, high energy density, and extensive use of additives. Epidemiological data suggest an association between higher UPF intake and adverse renal outcomes, yet the underlying mechanisms remain insufficiently defined. We posit inflammaging, a chronic, low-grade inflammatory state linked to biological aging, as a conceptual framework through which UPF-related renal injury may be interpreted. Within this context, gut dysbiosis and excess dietary phosphate emerge as potential mediators. Although no causal relationship has been established until now, there is mounting evidence interconnecting UPF’s consumption, hidden dietary phosphorus, chronic low-grade inflammation, accelerated aging and gut dysbiosis with CKD progression. We highlight critical research gaps and emphasize the need for policy and population-level strategies to reduce UPF consumption and slow CKD progression.

## 1. Introduction

**Figure 1:**
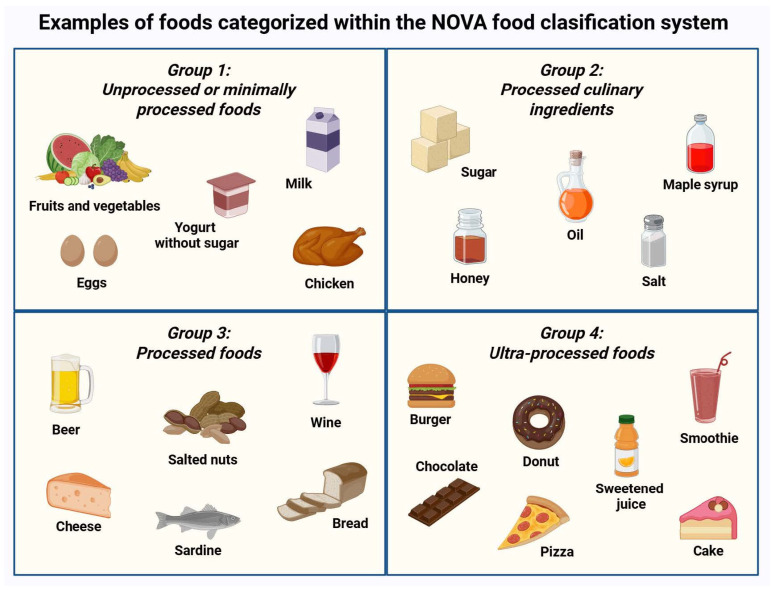
NOVA food classification system. Foods are grouped into four categories according to the extent and purpose of processing. Representative examples of UPFs (group 4) include burgers, cakes, and sweetened fruit drinks or smoothies. Created in BioRender. Kounatidis, D. (2026) https://BioRender.com/dr5fznn (assessed on 25 March 2026).

Ultra-processed foods (UPFs) are industrial formulations classified by the NOVA system as the most extensively modified category of foods. These products undergo multiple physical and chemical transformations that substantially alter the native food matrix. As a result, UPFs are typically energy-dense and nutritionally imbalanced, providing high amounts of added sugars, sodium, and refined fats, while being depleted of dietary fiber, vitamins, and bioactive compounds. In addition, a wide range of additives, including artificial sweeteners, emulsifiers, and colorants, are used to enhance palatability and shelf life. Food processing and packaging may also introduce chemical contaminants, including endocrine-disrupting chemicals (EDCs), with potential noxious biological effects [1,2,3]. Figure 1 presents examples of foods classified within the NOVA system.

Chronic kidney disease (CKD), defined by a sustained reduction in glomerular filtration rate (GFR) and/or persistent albuminuria for at least three months, affects more than 850 million individuals worldwide and is projected to become the fifth leading cause of death by 2050 [4]. The burden is particularly high in low- and middle-income countries, where limited screening, delayed diagnosis, and restricted access to nephroprotective therapies contribute to faster progression to end-stage renal disease (ESRD) [5]. Diabetes and hypertension remain the principal causes of CKD, with mounting evidence implicating UPF-rich diets as contributors, yet the underlying mechanisms remain under investigation [6,7].

This narrative review examines the relationship between UPF-related renal injury and proposes inflammaging, a chronic low-grade inflammatory state that intensifies with age, as a potential mechanistic link. Gut dysbiosis and high dietary phosphorus, both associated with UPF consumption, may exacerbate this process by sustaining inflammation and accelerating pro-aging pathways that drive CKD onset and progression. We also identify critical gaps in current knowledge and discuss public health strategies to mitigate the growing global burden of CKD in populations increasingly reliant on ultra-processed diets.

## 2. Clinical Data Linking Ultra-Processed Foods to Chronic Kidney Disease

In recent years, a growing body of clinical research has supported that UPF-rich diets are associated with both the development and progression of CKD. Early indications emerged from the Chronic Renal Insufficiency Cohort (CRIC) Study, a large U.S. prospective cohort designed to examine determinants of CKD progression. In an analysis including 3939 adults with established early-stage CKD enrolled between 2003 and 2008, Sullivan and colleagues assessed dietary intake using food frequency questionnaires (FFQs). Participants with greater consumption of ultra-processed products experienced a significantly faster transition to advanced stages of kidney disease and exhibited higher all-cause mortality, even after adjustment for major cardiometabolic risk factors [8]. These findings suggest that, beyond traditional nutrient-based indicators, the degree of industrial food processing may represent an independent dimension of dietary risk in individuals already living with impaired renal function. Similar patterns have been observed in Asian populations. In a Taiwanese cohort of 41,128 middle-aged and older adults monitored between 2008 and 2010, Kurniawan et al. examined participants with reduced renal reserve, defined by a baseline estimated glomerular filtration rate (eGFR) below 90 mL/min/1.73 m^2^ accompanied by albuminuria. Frequent consumption of processed meats and instant noodles was associated with progression to moderate or severe CKD, whereas dietary patterns rich in plant-based foods were linked to a slower decline in renal function [9].

Comparable observations have been reported in long-term population cohorts. In the Nurses’ Health Study, Lin et al. analyzed data from 3071 older women, who were followed between 1989 and 2000, incorporating repeated measurements of eGFR and urinary albumin-to-creatinine ratio (ACR). A Western dietary pattern, characterized by high UPF intake, was associated with a more pronounced decline in eGFR and worsening albuminuria over time. In contrast, adherence to the Dietary Approaches to Stop Hypertension (DASH) dietary pattern correlated with slower deterioration of kidney function, highlighting the potential benefits of dietary models centered on minimally processed foods [10]. Long-term prospective evidence also comes from the Atherosclerosis Risk in Communities (ARIC) Study. Du et al. evaluated 14,679 middle-aged adults with preserved kidney function at baseline (1987–1989). Over more than three decades of follow-up, 4859 participants developed CKD. Higher baseline consumption of UPFs was independently associated with an increased risk of incident CKD, providing longitudinal support that the degree of food processing may influence the initiation of renal dysfunction [11].

These epidemiological signals are supported by recent meta-analyses. He et al. reported that each 10% increase in the dietary contribution of UPFs was associated with an approximately 7% higher risk of CKD, consistent with a linear dose–response relationship [12]. Similarly, Xiao et al. found that individuals with the highest UPF intake had a significantly greater risk of CKD compared with those consuming the lowest amounts [13]. Table 1 summarizes key observational studies examining the association between UPF intake and CKD.

Despite accumulating epidemiological evidence, the biological pathways linking ultra-processed dietary exposures to renal injury remain insufficiently characterized. The following sections examine this relationship, with emphasis on chronic low-grade inflammation and accelerated biological aging as potential mediators.

## 3. Inflammation and Aging in Chronic Kidney Disease

### 3.1. Main Inflammatory Pathways

CKD unfolds within a chronic state of low-grade inflammation that progressively impairs renal architecture and function. Within the kidney, tissue-resident and infiltrating macrophages act as central regulators of innate immune activity, coordinating inflammatory responses in the local microenvironment. Macrophages express a diverse array of pattern-recognition receptors (PRRs), including Toll-like receptors (TLRs). These receptors detect and respond to pathogen-associated molecular patterns (PAMPs) and damage-associated molecular patterns (DAMPs), such as lipopolysaccharide (LPS). Engagement of these receptors, together with signaling initiated by cytokine receptors such as those for tumor necrosis factor-α (TNF-α) and interleukin-1β (IL-1β), activates intracellular kinase cascades that converge on nuclear factor κB (NF-κB) signaling. Phosphorylation and subsequent degradation of the inhibitor of κB (IκB) releases NF-κB from cytoplasmic sequestration, enabling its translocation to the nucleus and transcriptional activation of pro-inflammatory cytokines, chemokines and adhesion molecules. Through autocrine amplification of macrophage activation and paracrine recruitment of additional immune cells, these mediators establish a persistent inflammatory niche within renal tissue [16,17].

Inflammatory signaling in CKD is closely intertwined with oxidative stress pathways. Sustained NF-κB activation can attenuate the activity of nuclear factor erythroid 2–related factor 2 (Nrf2), the principal regulator of cellular antioxidant defense systems. Crosstalk between these pathways occurs through modulation of the Kelch-like ECH-associated protein 1 (Keap1)–Nrf2 regulatory axis and competition for transcriptional co-activators, ultimately reducing the expression of cytoprotective and detoxifying enzymes. As antioxidant capacity declines, reactive oxygen species (ROS) and reactive nitrogen species (RNS) accumulate, promoting lipid peroxidation, protein carbonylation and the formation of advanced glycation end products (AGEs). Many of these oxidative modifications function as DAMPs, reinforcing PRR-mediated signaling and thereby facilitating inflammatory circuits that contribute to progressive renal injury [17,18].

A further escalation of inflammatory activity occurs with activation of the NLR family pyrin domain-containing 3 (NLRP3) inflammasome. Assembly of this multiprotein complex requires two sequential signals. The initial priming step is driven by PRR-dependent NF-κB activation, which upregulates transcription of NLRP3 together with the inactive cytokine precursors pro-IL-1β and pro-IL-18. A second signal arises from cellular stress and metabolic perturbations, most commonly triggered by adenosine triphosphate (ATP)-mediated potassium efflux, mitochondrial dysfunction and lysosomal destabilization. Damaged mitochondria generate excessive mitochondrial ROS and release oxidized mitochondrial DNA (mtDNA) into the cytosol, where it acts as a potent DAMP. At the same time, redistribution of cardiolipin from the inner to the outer mitochondrial membrane promotes direct interaction with NLRP3, facilitating inflammasome assembly, whereas lysosomal rupture provides an additional activating signal through the cytosolic release of cathepsins [19,20,21].

**Figure 2:**
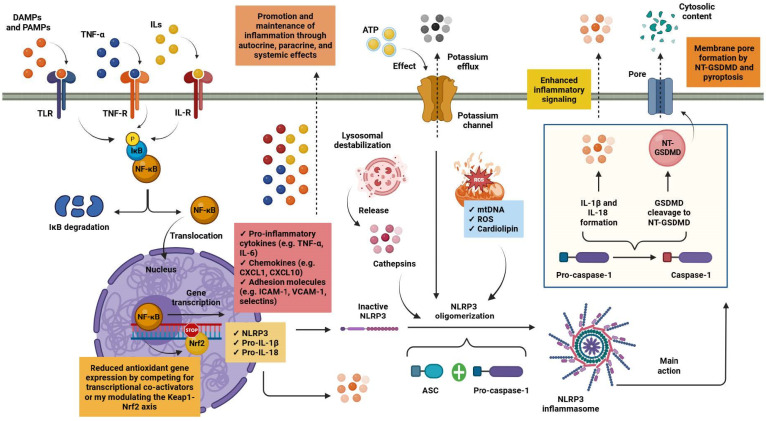
Inflammatory circuits in chronic kidney disease pathogenesis and progression. Tissue-resident and infiltrating macrophages act as key regulators of innate immune responses via TLRs that sense PAMPs and DAMPs. Activation of these pathways, together with TNF-α and IL-1β signaling, leads to NF-κB activation, inducing the transcription of pro-inflammatory mediators that enhance immune cell recruitment and sustain macrophage activation. Persistent NF-κB signaling suppresses Nrf2 activity, impairing antioxidant defenses and enhancing oxidative stress. Concurrently, NF-κB primes inflammasome activation by upregulating NLRP3 and pro-inflammatory cytokine precursors. Secondary danger signals, including potassium efflux, lysosomal disruption, and mitochondrial dysfunction, trigger NLRP3 inflammasome assembly through recruitment of ASC and pro-caspase-1. Inflammasome activation results in caspase-1–dependent maturation of IL-1β and IL-18 and GSDMD–mediated pyroptosis, thereby amplifying inflammation and promoting renal injury. Abbreviations: ASC: apoptosis-associated speck-like protein containing a caspase recruitment domain; ATP: adenosine triphosphate; CXCL1: C-X-C motif chemokine ligand 1; CXCL10: C-X-C motif chemokine ligand 10; DAMPs: damage-associated molecular patterns; GSDMD: gasdermin-D, IκΒ: inhibitor of κΒ; ICAM-1: intercellular adhesion molecule 1; IL: interleukin; IL-R: interleukin receptor; mtDNA: mitochondrial DNA; NF-κB: nuclear factor κB; Nrf2: nuclear factor erythroid 2–related factor 2; NLRP3: NLR family pyrin domain containing 3; NT-GSDMD: N-terminal GSDMD; PAMPs: pathogen-associated molecular patterns; ROS: reactive oxygen species; TLR: Toll-like receptor; TNF-α: tumor necrosis factor-alpha; TNF-R: tumor necrosis factor receptor; VCAM-1: vascular cell adhesion molecule 1. Created in BioRender. Kounatidis, D. (2026) https://BioRender.com/7sf103k (assessed on 25 March 2026).

Integration of these signals promotes oligomerization of NLRP3 and recruitment of the adaptor apoptosis-associated speck-like protein containing a caspase recruitment domain (ASC) together with pro-caspase-1. Within this complex, caspase-1 undergoes autocatalytic activation and cleaves pro-IL-1β and pro-IL-18 into their mature inflammatory forms. Activated caspase-1 also processes gasdermin D (GSDMD), generating an N-terminal fragment that inserts into the plasma membrane to form pores. This process initiates pyroptosis, a lytic form of programmed cell death characterized by the release of intracellular alarmins and additional DAMPs. The resulting enhancement of innate immune signaling perpetuates the inflammatory milieu that typifies CKD and contributes to progressive renal tissue injury [19,20,21]. Figure 2 depicts the main inflammatory pathways implicated in the pathogenesis and progression of CKD.

### 3.2. From Aging to Inflammaging

Aging is a complex biological process marked by a gradual loss of physiological resilience across organs and tissues, resulting from the cumulative accumulation of cellular and molecular damage over lifetime. This deterioration reflects the progressive dysregulation of multiple pathways, including genomic instability, telomere shortening, epigenetic modifications, mitochondrial dysfunction, impaired proteostasis, and defective autophagy. Together, these alterations compromise cellular homeostasis and progressively weaken tissue maintenance. Aging is also associated with stem cell depletion and disrupted intercellular communication, which further diminish tissue integrity and regenerative potential [22,23].

A central hallmark of aging is cellular senescence, a state of essentially irreversible cell-cycle arrest accompanied by extensive metabolic, transcriptional, and phenotypic reprogramming [24]. Senescent cells, although no longer capable of proliferation, remain metabolically active and adopt a distinct secretory profile, known as the senescence-associated secretory phenotype (SASP). This program includes a broad array of pro-inflammatory cytokines, chemokines, growth factors, and matrix-remodeling enzymes that reshape the local tissue microenvironment. Through sustained SASP secretion, senescent cells induce paracrine senescence in neighboring cells, modify extracellular matrix architecture, and reinforce a persistent pro-inflammatory milieu. The progressive accumulation of senescent cells, together with their altered secretory activity and reduced regenerative potential, is increasingly recognized as a major driver of tissue dysfunction and the onset of age-related diseases [25,26].

Aging is also accompanied by a progressive decline in immune competence, a phenomenon collectively termed immunosenescence. This process encompasses both quantitative and functional changes across adaptive and innate immune compartments, resulting in impaired immune surveillance, reduced pathogen clearance, and dysregulated inflammatory responses. In the adaptive immune system, thymic involution leads to a marked reduction in naïve T cell output, alongside expansion of memory and terminally differentiated T cell populations. These shifts are often accompanied by loss of the co-stimulatory molecule CD28 on T lymphocytes, reflecting replicative exhaustion and diminished proliferative capacity [27,28]. Innate immune cells undergo substantial remodeling with age as well. Natural killer (NK) cells exhibit shifts between CD56^bright^ and CD56^dim^ subsets, altering CD16 expression and their cytokine production and cytotoxic functions, potentially compromising NK-mediated immune surveillance. Dendritic cells (DCs) similarly experience functional decline, with myeloid DCs displaying impaired antigen presentation and modified responsiveness to PAMPs and DAMPs. Moreover, aged DCs adopt a pro-inflammatory bias, producing elevated levels of cytokines such as IL-6 and TNF-α [29,30].

**Figure 3:**
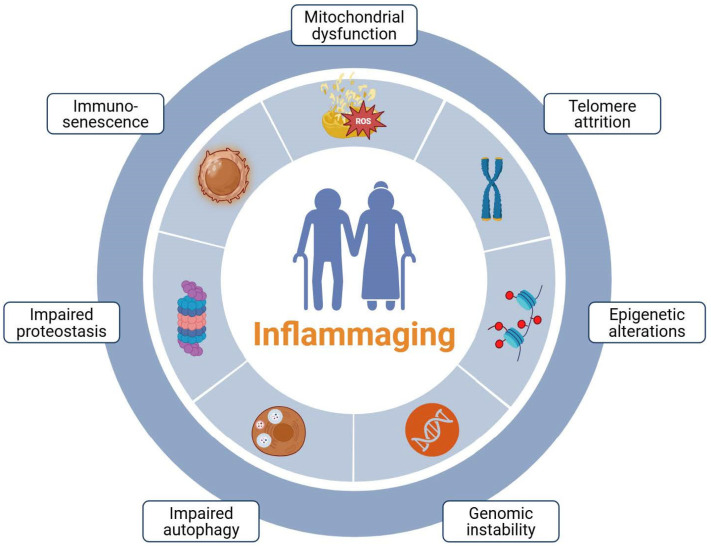
Key mediators driving inflammaging. Multiple biological aging processes, such as mitochondrial dysfunction, telomere shortening, and defective autophagy, can contribute to the development of inflammaging, which is implicated in age-related diseases, including chronic kidney disease. Created in BioRender. Kounatidis, D. (2026) https://BioRender.com/toemm31 (assessed on 25 March 2026).

Within this biological context, the concept of inflammaging, first introduced by Claudio Franceschi in 2000, describes the chronic, low-grade inflammatory state that develops during aging in the absence of overt infection. Inflammaging results from cumulative activation of innate and adaptive immune pathways and is closely linked to both immunosenescence and the progressive accumulation of senescent cells. Multiple mechanisms converge to sustain this pro-inflammatory milieu, including persistent SASP secretion, mitochondrial dysfunction, oxidative stress, and the accumulation of DAMPs. Persistent inflammatory signaling drives tissue injury, metabolic dysregulation, and progressive organ dysfunction, thereby heightening susceptibility to a broad spectrum of age-related diseases [31,32]. Figure 3 presents the principal drivers of inflammaging.

Organs with high metabolic demand and intricate microvascular architecture, such as the kidney, are particularly susceptible to the cumulative effects of inflammaging. Both innate and adaptive immune mechanisms contribute to CKD pathophysiology, linking systemic inflammatory aging with progressive renal injury [33]. Patients with advanced CKD, especially those with ESRD, frequently display features of premature immunological aging. This phenotype is characterized by reductions in naïve T cells alongside expansion of highly differentiated or senescent T cell populations [34]. Persistent inflammatory and oxidative signaling ultimately drives adverse structural remodeling in the kidney, including glomerulosclerosis, tubular atrophy, thickening of the glomerular basement membrane, and expansion of interstitial fibrosis [33,35].

## 4. Ultra-Processed Foods and Inflammaging

### 4.1. Ultra-Processed Foods and Inflammation

Mounting epidemiological evidence links dietary patterns rich in UPFs with inflammatory dysregulation. In a cross-sectional study of 1986 adults aged 46 to 70 years, Millar et al. reported that higher UPF intake was associated with increased circulating concentrations of IL-6 and TNF-α, elevated total leukocyte counts, and a higher neutrophil-to-lymphocyte ratio. Part of this relationship likely reflects the greater prevalence of adiposity among individuals with high UPF consumption, consistent with the fundamental role of excess adipose tissue as a source of pro-inflammatory cytokines [36]. However, the inflammatory signal cannot be attributed to adiposity alone, since other data support that associations between UPF intake and inflammatory markers persist after adjustment for body mass index (BMI) and other metabolic confounders [37,38]. Age-dependent effects have also been described, particularly in children, where the association between UPF intake and circulating IL-6 appears to become evident from around nine years of age [39].

Numerous studies have linked the macronutrient composition of UPFs to insulin resistance, inflammation, and overall metabolic dysregulation, with the formation of AGEs playing a vital role. Nevertheless, the pro-inflammatory impact of UPFs cannot be explained solely by their macronutrient profile [40]. This limitation has redirected focus toward non-nutritional constituents introduced during industrial processing. Among these, EDCs such as bisphenol A (BPA) and phthalates have emerged as key candidates. Experimental data indicate that these compounds perturb cellular redox homeostasis and immune function through tightly interconnected mechanisms. In particular, they enhance the generation of ROS, interfere with intracellular signaling networks, and modulate immune cell dynamics by influencing both proliferation and apoptotic pathways. Sustained oxidative stress, in turn, impairs Nrf2-mediated antioxidant defenses, creating a permissive environment for persistent inflammatory activation [41].

### 4.2. Ultra-Processed Foods and Biological Aging

Diets dominated by UPFs may also influence biological aging trajectories. Analysis of more than 16,000 adults from the National Health and Nutrition Examination Survey (NHANES) between 2003 and 2010 demonstrated that each 10% increase in energy intake from UPFs corresponded to an estimated 0.21-year increase in biological age. Individuals in the highest consumption category, with UPFs contributing 68 to 100% of total energy intake, exhibited biological ages approximately 0.9 years greater than those with minimal intake, even after accounting for overall diet quality and other confounders [42].

Among the mechanisms proposed, alterations in telomere dynamics have received particular attention. Data from the Seguimiento Universidad de Navarra (SUN) Project, involving nearly 900 older adults, revealed significantly shorter leukocyte telomeres among individuals consuming more than three daily servings of UPFs [43]. Comparable findings have been reported in middle-aged and older Korean populations, where higher intake of sugar-sweetened beverages was associated with reduced telomere length [44]. More recently, Rodrigues et al. highlighted epigenetic remodeling as a potential mechanism through which highly processed dietary exposures may influence aging, primarily via alterations in DNA methylation patterns [45]. Consistent with their role in inflammatory processes, EDCs appear to be key mediators in the association between UPF exposure and biological aging [46]. At the cellular level, BPA, even at low concentrations, has been shown to compromise genomic integrity, disrupt mitochondrial function, and induce structural alterations in cellular architecture [47]. Phthalates have been linked to telomere attrition early in life, whereas acrylamide generated during high-temperature processing promotes cellular senescence and endothelial dysfunction in experimental models [48,49].

Taken together, these observations support a multifactorial model in which the biological impact of UPFs reflects the combined influence of their nutritional profile and chronic exposure to processing-derived chemical agents. Through the convergence of these pathways, UPFs may potentiate inflammatory aging processes and increase susceptibility to chronic diseases, including CKD. Table 2 summarizes key epidemiological studies highlighting the contribution of UPF consumption to inflammatory processes and the acceleration of biological aging.

## 5. Microbiome Alterations

### 5.1. Ultra-Processed Foods, Chronic Kidney Disease, and the Emergence of Gut Dysbiosis

The human microbiome, comprising the collective genomes of bacteria, viruses, fungi, and archaea, resides predominantly within the gastrointestinal tract, functioning as a metabolic, immunological, and structural extension of the host. Under normal conditions, host tissues and resident microbial communities exist in a tightly regulated equilibrium that supports nutrient metabolism, immune maturation, and epithelial barrier integrity. Disruption of this balance, driven by genetic susceptibility or environmental exposures, can give rise to gut dysbiosis. This state is characterized by alterations in microbial composition, reduced taxonomic diversity, and shifts in microbial metabolic output [50].

Among environmental determinants, early-life antibiotic exposure and sedentary lifestyle are well-recognized contributors to microbial disruption. UPFs may further aggravate these disturbances, particularly by compromising the structural and functional integrity of the intestinal epithelial barrier [51]. This barrier relies on specialized intercellular complexes known as tight junctions (TJs), composed primarily of claudins, occludins, and junctional adhesion molecules (JAMs), which seal the paracellular space between epithelial cells. These multiprotein assemblies regulate selective diffusion of ions and small solutes while preventing the translocation of luminal antigens, microorganisms, and potentially harmful metabolites [50,52].

Evidence indicates that several additives commonly found in UPFs, including emulsifiers, artificial sweeteners, and certain food colorants, can disrupt the molecular organization and stability of TJs. Such perturbations weaken epithelial cohesion, reduce mucus-layer thickness, and compromise barrier defenses. The resulting increase in intestinal permeability, often referred to as “leaky gut,” facilitates systemic translocation of microbial components and metabolites originating from the intestinal lumen. This, in turn, triggers persistent inflammatory signaling, sustained immune cell recruitment, and chronic stress on key regulators of mucosal integrity, including epithelial cells, intestinal stem cells, and Paneth cells. Over time, these pressures can impair epithelial metabolism and regenerative capacity, accelerating functional decline of the intestinal barrier [50,52].

In individuals with CKD, a compromised barrier assumes particular pathological relevance. Increased intestinal permeability enhances systemic exposure to microbiota-derived uremic solutes, including indoxyl sulfate (IS), p-cresyl sulfate (PCS), and trimethylamine N-oxide (TMAO) [53]. TMAO is generated in the liver via oxidation of trimethylamine (TMA), a microbial metabolite produced during the intestinal metabolism of dietary substrates such as L-carnitine, choline, and phosphatidylcholine. Elevated circulating concentrations of these compounds reflect substantial microbial metabolic alterations and have been implicated in multiple pathogenic processes, including systemic inflammation, oxidative stress, endothelial dysfunction, and progressive renal injury [54].

**Figure 4:**
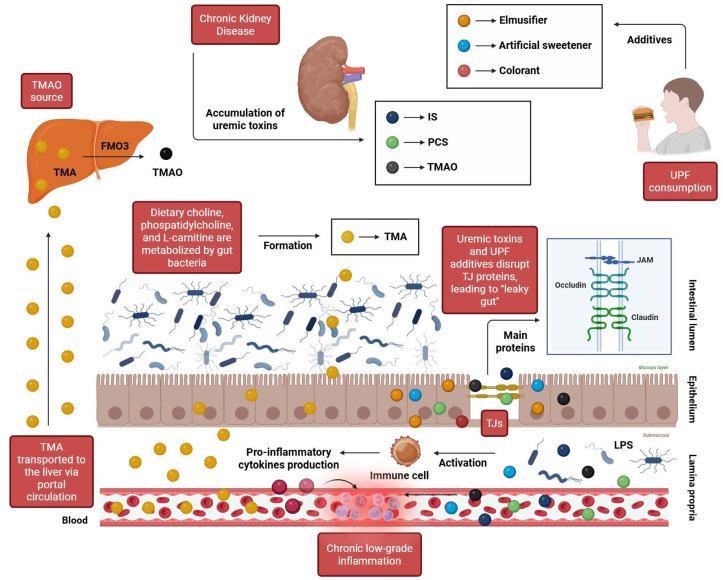
Key mechanisms linking ultra-processed foods and chronic kidney disease to intestinal barrier dysfunction. Ultra-processed foods disrupt tight junctions (claudins, occludins, JAMs) via additives such as emulsifiers, artificial sweeteners, and colorants, thereby weakening epithelial cohesion and reducing mucus thickness. In CKD, this “leaky gut” allows systemic translocation of microbiota-derived uremic toxins (IS, PCS, TMAO), favoring progressive renal injury. Abbreviations: FMO3: flavin-containing monooxygenase 3; IS: indoxyl sulfate; JAMs: junctional adhesion molecules; LPS: lipopolysaccharide; PCS: p-cresyl sulfate; TJs: tight junctions; TMA: trimethylamine; TMAO: trimethylamine N-oxide; UPFs: ultra-processed foods. Created in BioRender. Kounatidis, D. (2026) https://BioRender.com/77bpp27 (assessed on March 2026).

Overall, these mechanisms establish a bidirectional pathogenic loop. CKD-associated metabolic disturbances promote microbial imbalance and enhance the generation of gut-derived uremic toxins, which in turn further compromise intestinal barrier integrity and amplify systemic inflammatory signaling. The combined influence of CKD-related metabolic alterations and diets rich in UPFs thus favors sustained microbial dysregulation, reinforcing a cycle that may accelerate renal functional decline. Figure 4 illustrates central pathways through which UPF consumption and CKD converge to promote gut dysbiosis.

### 5.2. Gut Dysbiosis and Inflammaging

Accumulating evidence supports the occurrence of age-related shifts in gut microbiota composition and diversity, although findings across studies remain partly conflicting [55]. For instance, while it is commonly assumed that *Akkermansia muciniphila* declines with advancing age, some studies report its enrichment in the gut microbiota of healthy, long-lived individuals [56]. Moreover, both the timing and extent of microbiome alterations appear to vary across studies. Claesson et al. demonstrated that older individuals harbor a distinct core microbiota compared with younger adults, characterized by increased abundance of *Bacteroides* species, altered distributions of *Clostridium* groups, shifts in phylum-level composition, and considerable inter-individual variability despite relative short-term stability [57]. In contrast, other data suggest that overall microbial composition remains relatively stable until later stages of life, with more pronounced alterations observed in centenarians. In these individuals, the microbiota is marked by restructuring within the phylum *Firmicutes*, enrichment of facultative anaerobes including pathobionts, and reduced abundance of *Faecalibacterium prausnitzii* and other taxa with established anti-inflammatory properties. Such changes have been proposed to contribute to the development of inflammaging [58]. It is noteworthy that Santos-Pujol et al. have very recently conducted a multi-omics study of a woman with the most extreme lifespan, i.e., 117 years. In their study, they compared the fecal microbiota composition of this woman using 16S rDNA with 445 samples from 250 women and 195 men aged 61 to 91 years old. They managed to reveal that this supercentenarian woman had a favorable microbiome profile when compared to the control group. More specifically, she had reduced levels of the pro-inflammatory *Clostridium* genus and more abundance of *Bifidobacterium* regarding the fecal microbiota composition [59]. With their outstanding study, Santos-Pugol et al. documented that this woman with the most extreme lifespan had a beneficial microbiome composition as well as a younger epigenetic age. Their study provides further insight into the centenarians and most probably supercentenarians gut microbiome, thus advocating that longevity may be associated with a beneficial gut microbiome [59]. In sharp contrast, inflammaging seems to be characterized by a distorted gut microbiome as preliminary results have shown. Advancing age has been further associated with substantial remodeling of gut microbiota function and its immunometabolic interactions. Notably, reductions in key butyrate-producing bacteria, including *Eubacterium rectale*, may lead to decreased butyrate availability and subsequent impairment of regulatory B cell responses. Aging also alters mucosal immune dynamics within the gut-associated lymphoid tissue, as reflected by reduced proliferative capacity of CD4^+^ T cells in Peyer’s patches and diminished abundance and functionality of lamina propria Th17 cells. Declines in secondary bile acids may further disrupt intestinal immune homeostasis by promoting Th17 polarization while weakening regulatory T cell-mediated immunosuppression [60]. Importantly, the relationship between biological aging and gut dysbiosis appears to be bidirectional, as the gut microbiome continuously interacts with both the intestinal mucosa and the systemic immune system—networks that are closely linked to the development of frailty [61].

Adiposity represents an additional biological context in which metabolic and immune alterations converge with processes resembling accelerated aging [62]. Within adipose tissue, the accumulation of senescent cells contributes to a pro-inflammatory milieu through the secretion of senescence-associated mediators that enhance systemic immune activation. Notably, the clearance of these cells using senolytic agents has been shown to attenuate inflammatory signaling [63]. These mechanisms are particularly relevant to kidney disease. Obesity is strongly linked to major drivers of CKD, including type 2 diabetes (T2D) and hypertension, and may also exert direct renal effects, contributing to obesity-related glomerulopathy [64]. Such interactions may be amplified in dietary contexts characterized by high UPF intake, which is commonly associated with increased adiposity and metabolic disruption [65]. In obesity-associated nephropathy, microbiota-derived signals may additionally intersect with vascular endothelial growth factor A (VEGFA)-related pathways, potentially sustaining inflammatory responses across both renal and systemic compartments [66].

Collectively, although the evidence remains at an early stage and definitive conclusions cannot yet be drawn, these findings suggest that gut dysbiosis-associated inflammaging may represent a critical mechanistic link between high UPF consumption and adverse renal outcomes.

## 6. Hidden Dietary Phosphate in Ultra-Processed Foods and Its Role in Renal Inflammaging

Phosphate is an essential mineral involved in numerous physiological processes, including nucleic acid synthesis, cellular energy metabolism, and skeletal mineralization. Dietary intake is the primary determinant of systemic phosphate exposure. Within human diets, phosphate exists in two main forms, namely organic phosphate naturally present in foods such as grains, vegetables, meats, and dairy products, and inorganic phosphate salts that are commonly added to processed foods as additives, preservatives, or stabilizing agents [67,68]. These forms differ substantially in bioavailability, since organic phosphate is only partially absorbed, with uptake influenced by the food matrix, whereas inorganic phosphate salts are highly soluble and almost completely absorbed in the intestine. Consequently, the high content of phosphate-based additives in UPFs may drive increased systemic phosphate exposure that is not adequately reflected by total dietary phosphorus intake. In this regard, the widespread consumption of carbonated beverages, as a hidden dietary source of phosphorus, should be pointed out [69,70].

Phosphate balance is normally maintained through a complex network involving the kidneys, bones, and gastrointestinal tract. Elevated circulating phosphate stimulates osteocytes to release fibroblast growth factor 23 (FGF-23), a hormone that, together with its co-receptor Klotho, reduces renal tubular phosphate reabsorption and enhances urinary phosphate excretion. This feedback system aligns renal elimination with intestinal absorption and skeletal storage, thereby preserving systemic phosphate homeostasis. However, the efficiency of this compensatory network declines as kidney function deteriorates. Reduced renal excretory capacity increases susceptibility to phosphate retention, leading to secondary hyperparathyroidism, accelerated bone turnover, and vascular mineral deposition [71,72,73,74]. Notably, FGF-23 levels often rise early in CKD, frequently preceding overt hyperphosphatemia, and elevated concentrations are independently associated with increased cardiovascular morbidity and mortality [75].

Beyond its classical role in mineral metabolism, phosphate excess can actively drive tissue injury through enhanced inflammatory signaling, oxidative stress, and vascular calcification [76,77]. Disruptions in phosphate homeostasis may also influence biological aging processes. Elevated extracellular phosphate enters cells via sodium-dependent transporters, thereby increasing mitochondrial membrane potential and promoting ROS generation. This, in turn, can induce DNA damage and activate p53-dependent cell-cycle arrest, ultimately triggering cellular senescence. In the Baltimore Longitudinal Study of Aging, Zampino et al. studied 669 adults with a mean age of 67 years. They examined mitochondrial oxidative capacity in skeletal muscles by phosphorus magnetic resonance spectroscopy and demonstrated that reduced mitochondrial oxidative capacity was related to increased inflammatory biomarkers, like IL-6, CRP and ESR (Erythrocyte Sedimentation Rate) [78]. However, studies directly associating excess serum phosphorus levels with telomere attrition are lacking, as they are really difficult to perform. Nevertheless, such studies could shed light on a potential association between phosphorus and the aging process.

Interventions that reduce phosphate availability, whether via genetic manipulation or dietary restriction, have been shown to mitigate these aging-like phenotypes. Although human evidence remains largely observational, inverse associations between circulating phosphate levels and mammalian lifespan support the biological plausibility of phosphate-driven aging mechanisms [79].

**Figure 5:**
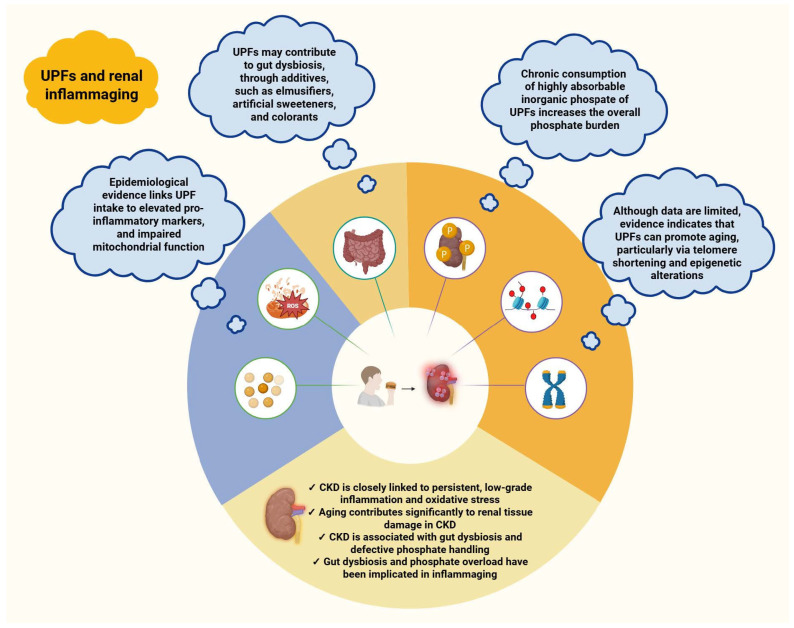
Suggested mechanisms linking ultra-processed foods to renal inflammaging. This relationship appears to involve aging–related mediators, including telomere shortening and epigenetic alterations, with additional contributions from gut dysbiosis and the hidden dietary phosphate content of UPFs. Abbreviations: CKD: chronic kidney disease; ROS: reactive oxygen species; UPFs: ultra-processed foods. Created in BioRender. Kounatidis, D. (2026) https://BioRender.com/el267p3 (assessed on 25 March 2026).

Taken together, these findings suggest that the hidden high-phosphate burden of ultra-processed dietary patterns may represent a subtle but significant pathway through which such diets promote renal inflammaging. Figure 5 summarizes the principal mechanisms by which UPFs may contribute to this process.

## 7. Research Gaps and Challenges

Apart from being rich in energy, trans-fatty acids and sodium, UPFs contain a plethora of additives, emulsifiers, flavor enhancers and colorants. Additionally, during the manufacturing and packaging process, other chemicals, such as phthalates, bisphenols, and polyfluoroalkyl substances (PFAS), may be produced. Although it is imperative to assess the generally recognized as safe (GRAS) ingredients as well as the estimated daily intake (EDI) in UPFs, this is not feasible. Indeed, the total dose of GRAS compounds cannot always be estimated due to the multiple sources of GRAS in UPFs. Furthermore, the GRAS substance refers to the dose and use of each and every compound and not within the context of multiple other substances consumed as well. Moreover, the EDI of chemical contaminants in UPFs remains largely unknown due to differences in the manufacturing processes and conditions. This fact poses a significant challenge regarding the study of UPFs [80].

Although clinical research linking UPF consumption with adverse kidney outcomes has expanded, the overall strength of the evidence remains limited. The available data are predominantly observational, with heterogeneous effect estimates and susceptibility to residual confounding, as noted in several meta-analyses [81]. Randomized controlled trials remain notably scarce due to ethical, methodological and financial factors [1]. Notably, short-term interventions suggest that diets based on minimally processed foods may reduce inflammatory markers such as high-sensitivity C-reactive protein (hs-CRP), whereas UPF-rich diets do not elicit a corresponding increase [82]. While informative, these findings are insufficient to establish causality, particularly in relation to long-term renal outcomes. Similar uncertainty surrounds the role of gut dysbiosis, as most evidence derives from preclinical models with unclear translational relevance. Nevertheless, a recent two-sample Mendelian randomization analysis points towards a potential causal contribution of specific microbial taxa, such as *Streptococcus*, to accelerated biological aging [83].

At the experimental level, the field remains narrowly focused on the effects of UPFs on selected pro-inflammatory markers. It is still unclear whether UPFs exert direct immunostimulatory effects or act indirectly through the accumulation of metabolic disturbances that amplify inflammatory signaling over time. In this context, the near absence of direct evidence linking UPF exposure to activation of the NLRP3 inflammasome is notable. This gap persists despite prior evidence showing that certain food-related compounds, such as titanium dioxide, previously used as a whitening agent in processed products, can induce NLRP3 activation and increase ROS production in inflammatory bowel disease [84]. Similar limitations extend to the study of biological aging pathways, where the evidence remains fragmented and largely exploratory. These gaps are particularly pronounced in CKD, where mechanistic studies examining how UPFs may contribute to renal vulnerability are largely lacking.

Despite these gaps, the convergence of epidemiological associations with emerging experimental evidence supports inflammaging as a biologically plausible link between UPF consumption and CKD. This hypothesis is further supported by comparable findings in major CKD risk factors, including T2D, hypertension, and obesity, where UPF intake has been associated with adverse outcomes [85,86]. In 2023, Lopez-Otin et al. have defined twelve features comprising the phenomenon of aging: telomeres’ attrition, DNA instability, epigenetic alterations, mitochondrial dysfunction, cellular senescence, modifications in intercellular communications, stem cell exhaustion, disturbances in autophagy, loss of proteostasis, disabled nutrient-sensing, gut dysbiosis and chronic inflammation [87]. Even though there is not enough evidence that all of the aforementioned features hold true for UPFs and CKD, most of them seem to be present. Moreover, as Lopez-Otin et al. point out, these features are tightly interconnected and create a vicious cycle that could however be overcome with specific therapeutic interventions [87]. In this context, chronic low-grade inflammation and biological aging may serve as key mediators underpinning the association between increased consumption of UPFs and CKD.

## 8. Policy Measures and Translational Priorities

UPFs account for nearly 60% of total energy intake in high-income countries such as the United States and the United Kingdom, reflecting a substantial shift in dietary patterns with important cardiometabolic implications [87,88]. These particularly concerning trends have prompted dietary guidelines to emphasize the need to reconfigure food environments, prioritizing reductions in population-level UPF consumption alongside improvements in the availability and affordability of minimally processed foods [89,90]. This approach is particularly critical in CKD, a condition associated with substantial morbidity and mortality, largely driven by its heightened cardiovascular risk [91].

A key regulatory gap lies in the limited transparency of UPF labeling, particularly regarding the disclosure of phosphate-containing additives. Hidden dietary phosphate may adversely influence both CKD progression and related comorbidities, like T2D [92]. Addressing this gap requires targeted regulatory action, including mandatory and standardized labeling of additives with renal and metabolic relevance. Such measures would enable more precise clinical management while also supporting informed consumer decision-making. When implemented alongside structural policies that improve access to healthier alternatives, these interventions have the potential to mitigate the incidence and progression of CKD, as well as other diet-related chronic diseases [93,94,95].

Future research efforts should adopt a translational perspective, bridging mechanistic insights with clinical and population-level evidence. This includes the design of innovative trial methodologies capable of capturing long-term UPF effects, the integration of multi-omics approaches to elucidate biological pathways, and the incorporation of system-level analyses that account for the complex interplay between diet, metabolism, immunity, and aging. Through such a comprehensive and interdisciplinary framework, the field can advance toward more definitive conclusions and actionable interventions.

## 9. Conclusions

UPFs have become a dominant feature of contemporary diets, raising significant concerns regarding their long-term impact on human health. Emerging epidemiological evidence links high UPF consumption with both the development and progression of CKD, although the precise mechanisms remain incompletely defined. Considering the established roles of chronic inflammation and biological aging in CKD, alongside accumulating evidence that UPFs may modulate these processes, inflammaging appears to be a plausible mediator connecting UPF exposure to renal vulnerability. Additional factors, including gut dysbiosis and increased dietary phosphate intake, further highlight potential pathways through which UPFs may interact with inflammatory and aging-related mechanisms relevant to kidney dysfunction. Despite these insights, the current evidence base is predominantly observational, limiting the capacity to draw causal inferences and underscoring the need for well-designed randomized controlled trials. In the interim, the adoption of targeted policy measures represents a central strategy to reduce the population-level burden of CKD.

## Figures and Tables

**Table 1:** Major epidemiological studies linking ultra-processed foods to chronic kidney disease.

Author, Year	Study Population	Findings	Remarks
Sullivan et al., 2023 [8]	3939 patients in the United States in the CRIC Study were followed for a median of 7 years.	Patients in the highest tertile of consumption of UPFs had an increased risk of progression to moderate to severe CKD, when compared to patients in the lowest tertile [HR, 1.22; 95% CI, 1.04–1.42; *p* = 0.01].	The increased consumption of UPFs was associated with a higher all-cause mortality rate in patients with CKD.
Kurniawan et al., 2019 [9]	41,128 middle-aged and older participants in Taiwan were enrolled between 2008 and 2010.	Patients with increased consumption of processed meat and lower consumption of plant-based food were found to have more severe stages of CKD [OR = 1.15, 95% CI 1.02–1.29, *p* < 0.05].	The authors concluded that higher intake of processed meat and animal products and lower consumption of plant-based food were associated with more severe CKD.
Lin et al., 2011 [10]	3071 women in the Nurse’s Health Study in the United States were followed for 11 years.	Women at the highest quartile of UPFs consumption had a rapid increase in microalbuminuria [OR, 2.17; 95% CI, 1.18–3.66; p for trend = 0.01] and also an accelerated deterioration in eGFR, when compared with women at the lowest quartile [OR, 1.77; 95% CI, 1.03–3.03].	While a DASH diet may be protective against CKD, the Western diet, which is rich in UPFs, was associated with an increased risk of CKD.
Du et al., 2022 [11]	14,679 individuals in the ARIC Study in the United States were followed for a median of 24 years.	Participants in the highest quartile of consumption of UPFs had a 24% higher risk of developing CKD, when compared to participants in the lowest quartile [HR, 1.24 95% CI, 1.15–1.35].	An increased consumption of UPFs was associated with a higher risk of incident CKD in this population-based study.
Kityo et al., 2022 [14]	Data were collected among 134,544 participants in the HEXA Study (Health Examinees Study) in Korea between 2004 and 2013.	Participants at the highest quartile of consumption of UPFs had increased prevalence of CKD [PR, 1.16, 95% CI 1.07–1.25].	The authors concluded that increased intake of UPFs was related to a higher prevalence of CKD in this Korean population.
Haring et al., 2017 [15]	11,952 adults aged 44 to 66 y.o. in the ARIC Study in the United States were followed for a median of 23 years.	Among the 11,952 participants, 2632 individuals developed incident CKD. Individuals in the highest quintile of consumption of UPFs had an increased risk for CKD [HRQ5, when compared to Q1: 1.23, 95% CI: 1.06, 1.42, *p* = 0.01].	The consumption of processed meat was associated with development of incident CKD, whereas increased consumption of nuts, legumes and low-fat dairy products was found to be protective against CKD development.

Abbreviations: ARIC: Atherosclerosis Risk in Communities; CI: confidence interval; CKD: chronic kidney disease; CRIC: Chronic Renal Insufficiency Cohort; eGFR: estimated glomerular filtration rate; HR: hazard ratio; HRQ5: highest quintile (5th quintile); OR: odds ratio; PR: prevalence ratio; UPFs: ultra-processed foods.

**Table 2:** Summary of epidemiological studies linking ultra-processed foods to inflammation and biological aging acceleration.

Author, Year	Study Population	Findings	Conclusions
Millar et al., 2025 [36]	1986 middle- to older-aged men and women	✔ Higher UPF intake is associated with increased IL-6, and TNF-α levels, as well as elevated WBC counts and NLR ✔ Associations persisted after adjustment for confounders and additional control for BMI or WHtR ✔ Adiposity partially mediated the relationship (12.7–70.1% via BMI; 13.5–64.5% via WHtR)	UPF consumption is linked to a more pro-inflammatory profile. This relationship appears to arise both from greater adiposity associated with UPF intake and from the inherent inflammatory potential of these foods.
Rodrigues et al., 2025 [45]	✔ 30 women, divided into tertiles based on their UPF intake ✔ Epigenetic analysis conducted in 15 women from the lowest and highest UPF intake tertiles	✔ 80 differentially methylated regions detected between groups ✔ The majority of these regions were hypomethylated in the high-UPF group	Elevated UPF intake is linked to measurable epigenetic alterations, particularly shifts in DNA methylation patterns.
Awad et al., 2025 [39]	450 children aged 7–10 years from a birth cohort in Chile (2023)	✔ A significant trend was observed between UPF intake and IL-1β levels (p-trend = 0.01) ✔ Age-stratified analysis showed an association between UPF intake and IL-6 among children ≥ 9 years (interaction *p* = 0.02)	UPF consumption may be linked to altered cytokine levels in school-aged children, suggesting inflammation as a possible pathway through which these foods could affect health early in life.
Esposito et al., 2024 [46]	22,495 adults from the Moli-sani Study (Italy, 2005–2010)	✔ Highest UPF consumption is associated with accelerated biological aging (β = 0.34 years; 95% CI: 0.08–0.61) ✔ Association showed a non-linear pattern (p for nonlinearity = 0.049) ✔ Adjustment for Mediterranean Diet Score slightly attenuated the association (~9%)	Greater UPF consumption is linked to modest acceleration of biological aging. The relationship is only partly explained by poorer dietary quality, implying that non-nutritional properties of UPFs may contribute to aging-related biological changes.
Silva Dos Santos et al., 2023 [37]	524 adults from the EPITeen Cohort (Portugal) and 2888 adults from the 1982 Pelotas Birth Cohort (Brazil)	✔ EPITeen females: IL-6 rose from 1.31 to 2.64 pg/mL across UPF quartiles ✔ Pelotas males: IL-6 rose from 1.40 to 1.59 pg/mL across UPF quartiles ✔ Mediation analysis showed that body fat mass did not explain the association	Higher UPF consumption is linked to elevated IL-6 concentrations, indicating increased inflammation, independent of adiposity.
Alonso-Pedrero et al., 2020 [43]	886 adults (645 men, 241 women) aged 57–91 years from the SUN Project, Spain (1999–2018)	✔ Adjusted OR for shortest telomeres in highest vs. lowest UPF quartile: 1.82 (95% CI: 1.05–3.22; p-trend = 0.03)	Greater UPF intake (>3 servings/day) was associated with an increased risk of shorter telomeres in this elderly Spanish population.
Lee et al., 2015 [44]	1958 middle-aged and older Korean adults	✔ Prudent dietary pattern (whole grains, seafood, legumes, vegetables, seaweed) is positively associated with longer LTL after multivariable adjustment ✔ In the analysis of particular food items, higher intake of legumes, nuts, seaweed, and fruits was linked to longer LTL ✔ Lower intake of red meat or processed meat and sweetened carbonated beverages was associated with longer LTL	Dietary habits of approximately 10 years earlier may influence the degree of biological aging in middle-aged and older adults.
Cardoso et al., 2010 [42]	16,055 adults aged 20–79 years (51% women) from the NHANES 2003–2010	✔ Each 10% increase in UPF intake is associated with 0.21 years higher biological age (95% CI: 0.16–0.26) ✔ Highest UPF quintile (68–100%) is correlated with 0.86 years older vs. lowest quintile (≤39%) ✔ Healthy diet adherence only moderately reduces the association (β = 0.14 per 10% UPF)	Higher UPF consumption is linked to accelerated biological aging. This effect is partly independent of overall diet quality, suggesting that the level of food processing itself may contribute to faster aging.

Abbreviations: BMI: body mass index; CI: confidence interval; EPITeen: Epidemiological Health Investigation of Teenagers in Portugal; IL: interleukin; LTL: leukocyte telomere length; NHANES: National Health and Nutrition Examination Survey; NLR: neutrophil-to-lymphocyte ratio; OR: odds ratio; SUN: Seguimiento Universidad de Navarra; TNF-α: tumor necrosis factor-alpha; UPFs: ultra-processed foods; WBC: white blood cells; WHtR: waist-to-height ratio.

## Data Availability

No new data were created or analyzed in this study. Data sharing is not applicable to this article.

## Abbreviations

AGEs: advanced glycation end products; ARIC: Atherosclerosis Risk in Communities; ASC: apoptosis-associated speck-like protein containing a caspase recruitment domain; ATP: adenosine triphosphate; BMI: body mass index; BPA: bisphenol A; CI: confidence interval; CKD: chronic kidney disease; CRIC: Chronic Renal Insufficiency Cohort; CXCL1: C-X-C motif chemokine ligand 1; CXCL10: C-X-C motif chemokine ligand 10; DAMPs: damage-associated molecular patterns; DASH: Dietary Approaches to Stop Hypertension; DCs: dendritic cells; EDCs: endocrine-disrupting chemicals; eGFR: estimated glomerular filtration rate; EPITeen: Epidemiological Health Investigation of Teenagers in Portugal; ESRD: end-stage renal disease; FFQs: food frequency questionnaires; FGF-23: fibroblast growth factor 23; GFR: glomerular filtration rate; GSDMD: gasdermin D; HR: hazard ratio; HRQ5: highest quintile (5th quintile); hs-CRP: high-sensitivity C-reactive protein; ICAM-1: intercellular adhesion molecule 1; IL: interleukin; IL-1β: interleukin-1β; IL-R: interleukin receptor; IκB: inhibitor of κB; IS: indoxyl sulfate; JAMs: junctional adhesion molecules; Keap1: Kelch-like ECH-associated protein 1; LPS: lipopolysaccharide; LTL: leukocyte telomere length; mtDNA: mitochondrial DNA; NF-κB: nuclear factor κB; NHANES: National Health and Nutrition Examination Survey; NK: natural killer; NLR: neutrophil-to-lymphocyte ratio; NLRP3: NLR family pyrin domain containing 3; Nrf2: nuclear factor erythroid 2–related factor 2; NT-GSDMD: N-terminal GSDMD; OR: odds ratio; PAMPs: pathogen-associated molecular patterns; PCS: p-cresyl sulfate; PR: prevalence ratio; PRRs: pattern-recognition receptors; RNS: reactive nitrogen species; ROS: reactive oxygen species; SASP: senescence-associated secretory phenotype; SUN: Seguimiento Universidad de Navarra; T2D: type 2 diabetes; TJs: tight junctions; TLR: Toll-like receptor; TMA: trimethylamine; TMAO: trimethylamine N-oxide; TNF-α: tumor necrosis factor-alpha; TNF-R: tumor necrosis factor receptor; UPFs: ultra-processed foods; VCAM-1: vascular cell adhesion molecule 1; VEGFA: vascular endothelial growth factor A; WBC: white blood cells; WHtR: waist-to-height ratio.

## References

[1] Monteiro C.A., Louzada M.L., Steele-Martinez E., Cannon G., Andrade G.C., Baker P., Bes-Rastrollo M., Bonaccio M., Gearhardt A.N., Khandpur N. (2025). Ultra-processed foods and human health: The main thesis and the evidence. Lancet.

[2] Monteiro C.A., Cannon G., Levy R.B., Moubarac J.C., Louzada M.L., Rauber F., Khandpur N., Cediel G., Neri D., Martinez-Steele E. (2019). Ultra-processed foods: What they are and how to identify them. Public Health Nutr..

[3] Dalamaga M., Kounatidis D., Tsilingiris D., Vallianou N.G., Karampela I., Psallida S., Papavassiliou A.G. (2024). The Role of Endocrine Disruptors Bisphenols and Phthalates in Obesity: Current Evidence, Perspectives and Controversies. Int. J. Mol. Sci..

[4] Jager K.J., Kovesdy C., Langham R., Rosenberg M., Jha V., Zoccali C. (2019). A single number for advocacy and communication-worldwide more than 850 million individuals have kidney diseases. Nephrol. Dial. Transplant..

[5] Yan M.T., Chao C.T., Lin S.H. (2021). Chronic Kidney Disease: Strategies to Retard Progression. Int. J. Mol. Sci..

[6] Francis A., Harhay M.N., Ong A.C.M., Tummalapalli S.L., Ortiz A., Fogo A.B., Fliser D., Roy-Chaudhury P., Fontana M., Nangaku M. (2024). Chronic kidney disease and the global public health agenda: An international consensus. Nat. Rev. Nephrol..

[7] Leonberg K.E., Maski M.R., Scott T.M., Naumova E.N. (2025). Ultra-Processed Food and Chronic Kidney Disease Risk: A Systematic Review, Meta-Analysis, and Recommendations. Nutrients.

[8] Sullivan V.K., Appel L.J., Anderson C.A.M., Kim H., Unruh M.L., Lash J.P., Trego M., Sondheimer J., Dobre M., Pradhan N. (2023). CRIC Study Investigators. Ultraprocessed Foods and Kidney Disease Progression, Mortality, and Cardiovascular Disease Risk in the CRIC Study. Am. J. Kidney Dis..

[9] Kurniawan A.L., Hsu C.Y., Rau H.H., Lin L.Y., Chao J.C. (2019). Association of kidney function-related dietary pattern, weight status, and cardiovascular risk factors with severity of impaired kidney function in middle-aged and older adults with chronic kidney disease: A cross-sectional population study. Nutr. J..

[10] Lin J., Fung T.T., Hu F.B., Curhan G.C. (2011). Association of dietary patterns with albuminuria and kidney function decline in older white women: A subgroup analysis from the Nurses’ Health Study. Am. J. Kidney Dis..

[11] Du S., Kim H., Crews D.C., White K., Rebholz C.M. (2022). Association Between Ultraprocessed Food Consumption and Risk of Incident CKD: A Prospective Cohort Study. Am. J. Kidney Dis..

[12] He X., Zhang X., Si C., Feng Y., Zhu Q., Li S., Shu L. (2024). Ultra-processed food consumption and chronic kidney disease risk: A systematic review and dose-response meta-analysis. Front. Nutr..

[13] Xiao B., Huang J., Chen L., Lin Y., Luo J., Chen H., Fu L., Tang F., Ouyang W., Wu Y. (2024). Ultra-processed food consumption and the risk of incident chronic kidney disease: A systematic review and meta-analysis of cohort studies. Ren. Fail..

[14] Kityo A., Lee S.A. (2022). The Intake of Ultra-Processed Foods and Prevalence of Chronic Kidney Disease: The Health Examinees Study. Nutrients.

[15] Haring B., Selvin E., Liang M., Coresh J., Grams M.E., Petruski-Ivleva N., Steffen L.M., Rebholz C.M. (2017). Dietary Protein Sources and Risk for Incident Chronic Kidney Disease: Results From the Atherosclerosis Risk in Communities (ARIC) Study. J. Ren. Nutr..

[16] Ren N., Wang W.F., Zou L., Zhao Y.L., Miao H., Zhao Y.Y. (2024). The nuclear factor kappa B signaling pathway is a master regulator of renal fibrosis. Front. Pharmacol..

[17] Pedruzzi L.M., Stockler-Pinto M.B., Leite M., Mafra D. (2012). Nrf2-keap1 system versus NF-κB: The good and the evil in chronic kidney disease?. Biochimie.

[18] He F., Ru X., Wen T. (2020). NRF2, a Transcription Factor for Stress Response and Beyond. Int. J. Mol. Sci..

[19] Kounatidis D., Vallianou N., Evangelopoulos A., Vlahodimitris I., Grivakou E., Kotsi E., Dimitriou K., Skourtis A., Mourouzis I. (2023). SGLT-2 Inhibitors and the Inflammasome: What’s Next in the 21st Century?. Nutrients.

[20] Komada T., Muruve D.A. (2019). The role of inflammasomes in kidney disease. Nat. Rev. Nephrol..

[21] Huang G., Zhang Y., Zhang Y., Ma Y. (2022). Chronic kidney disease and NLRP3 inflammasome: Pathogenesis, development and targeted therapeutic strategies. Biochem. Biophys. Rep..

[22] da Costa J.P., Vitorino R., Silva G.M., Vogel C., Duarte A.C., Rocha-Santos T. (2016). A synopsis on aging-Theories, mechanisms and future prospects. Ageing Res. Rev..

[23] Tartiere A.G., Freije J.M.P., López-Otín C. (2024). The hallmarks of aging as a conceptual framework for health and longevity research. Front. Aging.

[24] Maldonado E., Morales-Pison S., Urbina F., Solari A. (2023). Aging Hallmarks and the Role of Oxidative Stress. Antioxidants.

[25] Ajoolabady A., Pratico D., Bahijri S., Eldakhakhny B., Tuomilehto J., Wu F., Ren J. (2025). Hallmarks and mechanisms of cellular senescence in aging and disease. Cell Death Discov..

[26] Guo J., Huang X., Dou L., Yan M., Shen T., Tang W., Li J. (2022). Aging and aging-related diseases: From molecular mechanisms to interventions and treatments. Signal Transduct. Target Ther..

[27] Goyani P., Christodoulou R., Vassiliou E. (2024). Immunosenescence: Aging and Immune System Decline. Vaccines.

[28] Fu Y., Wang B., Alu A., Hong W., Lei H., He X., Shi H., Cheng P., Yang X. (2025). Immunosenescence: Signaling pathways, diseases and therapeutic targets. Signal Transduct. Target Ther..

[29] Guo Z., Wu F., Chen Y., Xu J., Chen Z. (2025). Phenotypes, mechanisms, and therapeutic strategies of natural killer cell immunosenescence. Immun. Ageing.

[30] Chidrawar S.M., Khan N., Chan Y.L., Nayak L., Moss P.A. (2006). Ageing is associated with a decline in peripheral blood CD56bright NK cells. Immun. Ageing.

[31] Fulop T., Larbi A., Pawelec G., Khalil A., Cohen A.A., Hirokawa K., Witkowski J.M., Franceschi C. (2023). Immunology of Aging: The Birth of Inflammaging. Clin. Rev. Allergy Immunol..

[32] Ferrucci L., Fabbri E. (2018). Inflammaging: Chronic inflammation in ageing, cardiovascular disease, and frailty. Nat. Rev. Cardiol..

[33] Yan J., Zhao F., Zhang R., Wu H., Lyu J., Shi L., Wang H. (2026). Renal inflammaging: Mechanisms, pathophysiology and therapeutic prospects. Ageing Res. Rev..

[34] Chiu Y.L., Shu K.H., Yang F.J., Chou T.Y., Chen P.M., Lay F.Y., Pan S.Y., Lin C.J., Litjens N.H.R., Betjes M.G.H. (2018). A comprehensive characterization of aggravated aging-related changes in T lymphocytes and monocytes in end-stage renal disease: The iESRD study. Immun. Ageing.

[35] Mihai S., Codrici E., Popescu I.D., Enciu A.M., Albulescu L., Necula L.G., Mambet C., Anton G., Tanase C. (2018). Inflammation-Related Mechanisms in Chronic Kidney Disease Prediction, Progression, and Outcome. J. Immunol. Res..

[36] Millar S.R., Harrington J.M., Perry I.J., Phillips C.M. (2025). Associations between ultra-processed food and drink consumption and biomarkers of chronic low-grade inflammation: Exploring the mediating role of adiposity. Eur. J. Nutr..

[37] Silva Dos Santos F., Costa Mintem G., Oliveira de Oliveira I., Lessa Horta B., Ramos E., Lopes C., Petrucci Gigante D. (2023). Consumption of ultra-processed foods and IL-6 in two cohorts from high- and middle-income countries. Br. J. Nutr..

[38] Mete B., Sadıkoğlu H.M., Demirhindi H., Melekoglu E., Barutcu A., Makca T., Atun Utuk F. (2024). The association between ultra-processed food consumption and low-grade inflammation in childhood: A cross-sectional study. Nutr. Bull..

[39] Awad C., Rubilar P., Hirmas-Adauy M., Iglesias V., Muñoz M.P., Retamal M.A., Carvajal C., Dadvand P., Lassale C. (2025). Ultra-Processed Foods and Markers of Systemic Inflammation in Children. Food Sci. Nutr..

[40] Mignogna C., Costanzo S., Di Castelnuovo A., Ruggiero E., Shivappa N., Hebert J.R., Esposito S., De Curtis A., Persichillo M., Cerletti C. (2022). The inflammatory potential of the diet as a link between food processing and low-grade inflammation: An analysis on 21,315 participants to the Moli-sani study. Clin. Nutr..

[41] Martínez Leo E.E., Peñafiel A.M., Hernández Escalante V.M., Cabrera Araujo Z.M. (2021). Ultra-processed diet, systemic oxidative stress, and breach of immunologic tolerance. Nutrition.

[42] Cardoso B.R., Liu J., Machado P., Kwon D., Belsky D.W., Martinez Steele E. (2024). Association between ultra-processed food intake and biological ageing in US adults: Findings from National Health and Nutrition Examination Survey (NHANES) 2003–2010. Age Ageing.

[43] Alonso-Pedrero L., Ojeda-Rodríguez A., Martínez-González M.A., Zalba G., Bes-Rastrollo M., Marti A. (2020). Ultra-processed food consumption and the risk of short telomeres in an elderly population of the Seguimiento Universidad de Navarra (SUN) Project. Am. J. Clin. Nutr..

[44] Lee J.Y., Jun N.R., Yoon D., Shin C., Baik I. (2015). Association between dietary patterns in the remote past and telomere length. Eur. J. Clin. Nutr..

[45] Rodrigues A.E., Fernandes A.E., Carrasco A.G.M., Pellenz F.M., da Rosa P.W.L., de Moura A.M.D.S.H., Santin F.G.O., Cercato C., de Melo M.E., Mancini M.C. (2025). High Consumption of Ultra-Processed Foods Is Associated with Genome-Wide DNA Methylation Differences in Women: A Pilot Study. Nutrients.

[46] Esposito S., Gialluisi A., Di Castelnuovo A., Costanzo S., Pepe A., Ruggiero E., De Curtis A., Persichillo M., Cerletti C., Donati M.B. (2024). Ultra-processed food consumption is associated with the acceleration of biological aging in the Moli-sani Study. Am. J. Clin. Nutr..

[47] Khan N.G., Tungekar B., Adiga D., Chakrabarty S., Rai P.S., Kabekkodu S.P. (2023). Alterations induced by Bisphenol A on cellular organelles and potential relevance on human health. Biochim. Biophys. Acta Mol. Cell Res..

[48] Song L., Liu B., Wu M., Zhang L., Wang L., Zhang B., Xiong C., Li Y., Cao Z., Wang Y. (2019). Prenatal Exposure to Phthalates and Newborn Telomere Length: A Birth Cohort Study in Wuhan, China. Environ. Health Perspect..

[49] Sellier C., Boulanger E., Maladry F., Tessier F.J., Lorenzi R., Nevière R., Desreumaux P., Beuscart J.B., Puisieux F., Grossin N. (2015). Acrylamide induces accelerated endothelial aging in a human cell model. Food Chem. Toxicol..

[50] Vallianou N.G., Kounatidis D., Panagopoulos F., Evangelopoulos A., Stamatopoulos V., Papagiorgos A., Geladari E., Dalamaga M. (2023). Gut Microbiota and Its Role in the Brain-Gut-Kidney Axis in Hypertension. Curr. Hypertens. Rep..

[51] Suliman I.L., Panculescu F.G., Fasie D., Cimpineanu B., Alexandru A., Gafar N., Popescu S., Nitu T.S., Enache F.D., Chisnoiu T. (2025). Gut Microbiome in Patients with Chronic Kidney Disease Stages 4 and 5: A Systematic Literature Review. Int. J. Mol. Sci..

[52] Anastasiou I.A., Kounatidis D., Vallianou N.G., Skourtis A., Dimitriou K., Tzivaki I., Tsioulos G., Rigatou A., Karampela I., Dalamaga M. (2025). Beneath the Surface: The Emerging Role of Ultra-Processed Foods in Obesity-Related. Cancer. Curr. Oncol. Rep..

[53] Rysz J., Franczyk B., Ławiński J., Olszewski R., Ciałkowska-Rysz A., Gluba-Brzózka A. (2021). The Impact of CKD on Uremic Toxins and Gut Microbiota. Toxins.

[54] Zhen J., Zhou Z., He M., Han H.X., Lv E.H., Wen P.B., Liu X., Wang Y.T., Cai X.C., Tian J.Q. (2023). The gut microbial metabolite trimethylamine N-oxide and cardiovascular diseases. Front. Endocrinol..

[55] Caldarelli M., Rio P., Marrone A., Giambra V., Gasbarrini A., Gambassi G., Cianci R. (2024). Inflammaging: The Next Challenge-Exploring the Role of Gut Microbiota, Environmental Factors, and Sex Differences. Biomedicines.

[56] Zeng S.Y., Liu Y.F., Liu J.H., Zeng Z.L., Xie H., Liu J.H. (2023). Potential Effects of *Akkermansia muciniphila* in Aging and Aging-Related Diseases: Current Evidence and Perspectives. Aging Dis..

[57] Claesson M.J., Cusack S., O’Sullivan O., Greene-Diniz R., de Weerd H., Flannery E., Marchesi J.R., Falush D., Dinan T., Fitzgerald G. (2011). Composition, variability, and temporal stability of the intestinal microbiota of the elderly. Proc. Natl. Acad. Sci. USA.

[58] Sena G., De Rango F., De Rose E., Perrotta A., Berardelli M., Scorza A., Cretella B., Passarino G., D’Aquila P., Bellizzi D. (2026). Composition of the Gut Microbiota in Older Adults Residing in a Nursing Home and Its Association with Dementia. Nutrients.

[59] Santos-Pujol E., Noguera-Castells A., Casado-Pelaez M., García-Prieto C.A., Vasallo C., Campillo-Marcos I., Quero-Dotor C., Crespo-García E., Bueno-Costa A., Setién F. (2025). The multi omics blueprint of the individual with the most extreme lifespan. Cell Rep. Med..

[60] Conway J., A Duggal N. (2021). Ageing of the gut microbiome: Potential influences on immune senescence and inflammaging. Ageing Res. Rev..

[61] Haran J.P., McCormick B.A. (2021). Aging, Frailty, and the Microbiome-How Dysbiosis Influences Human Aging and Disease. Gastroenterology.

[62] Santos A.L., Sinha S. (2021). Obesity and aging: Molecular mechanisms and therapeutic approaches. Ageing Res. Rev..

[63] Mau T., Yung R. (2018). Adipose tissue inflammation in aging. Exp. Gerontol..

[64] Kounatidis D., Vallianou N.G., Stratigou T., Voukali M., Karampela I., Dalamaga M. (2024). The Kidney in Obesity: Current Evidence, Perspectives and Controversies. Curr. Obes. Rep..

[65] Vallianou N.G., Kounatidis D., Tzivaki I., Zafeiri G.C.M., Rigatou A., Daskalopoulou S., Stratigou T., Karampela I., Dalamaga M. (2025). Ultra-Processed Foods and Childhood Obesity: Current evidence and perspectives. Curr. Nutr. Rep..

[66] Li J., Lv J.L., Cao X.Y., Zhang H.P., Tan Y.J., Chu T., Zhao L.L., Liu Z., Ren Y.S. (2022). Gut microbiota dysbiosis as an inflammaging condition that regulates obesity-related retinopathy and nephropathy. Front. Microbiol..

[67] Avesani C.M., Cardozo L.F.M.F., Yee-Moon Wang A., Shiels P.G., Lambert K., Lindholm B., Stenvinkel P., Mafra D. (2023). Planetary Health, Nutrition, and Chronic Kidney Disease: Connecting the Dots for a Sustainable Future. J. Ren. Nutr..

[68] Dunford E.K., Calvo M.S. (2025). Phosphate-based additives in processed foods: Is excess exposure a cause for concern? A cross-sectional examination of the United States packaged food supply. Am. J. Clin. Nutr..

[69] Rubio-Aliaga I. (2020). Phosphate and Kidney Healthy Aging. Kidney Blood Press. Res..

[70] Mafra D., Borges N.A., Lindholm B., Shiels P.G., Evenepoel P., Stenvinkel P. (2021). Food as medicine: Targeting the uraemic phenotype in chronic kidney disease. Nat. Rev. Nephrol..

[71] van Westing A.C., Küpers L.K., Geleijnse J.M. (2020). Diet and Kidney Function: A Literature Review. Curr. Hypertens. Rep..

[72] Chang A.R., Anderson C. (2017). Dietary Phosphorus Intake and the Kidney. Annu. Rev. Nutr..

[73] Nadkarni G.N., Uribarri J. (2014). Phosphorus and the kidney: What is known and what is needed. Adv. Nutr..

[74] Vogt J., Föller M. (2025). Regulation of αKlotho. Cell. Physiol. Biochem..

[75] Martínez-Heredia L., Canelo-Moreno J.M., García-Fontana B., Muñoz-Torres M. (2024). Non-Classical Effects of FGF23: Molecular and Clinical Features. Int. J. Mol. Sci..

[76] Yamada S., Tokumoto M., Tatsumoto N., Taniguchi M., Noguchi H., Nakano T., Masutani K., Ooboshi H., Tsuruya K., Kitazono T. (2014). Phosphate overload directly induces systemic inflammation and malnutrition as well as vascular calcification in uremia. Am. J. Physiol. Ren. Physiol..

[77] Ding M., Zhang Q., Zhang M., Jiang X., Wang M., Ni L., Gong W., Huang B., Chen J. (2022). Phosphate Overload Stimulates Inflammatory Reaction via PiT-1 and Induces Vascular Calcification in Uremia. J. Ren. Nutr..

[78] Zampino M., Brennan N.A., Kuo P.L., Spencer R.G., Fishbein K.W., Simonsick E.M., Ferrucci L. (2020). Poor mitochondrial health and systemic inflammation? Test of a classic hypothesis in the Baltimore Longitudinal Study of Aging. Geroscience.

[79] Jain N., Elsayed E.F. (2013). Dietary phosphate: What do we know about its toxicity. J. Nephrol..

[80] The FDA Guidance for Industry: Estimating Dietary Intake of Substances in Food. Issued by Human Foods Program 2006. https://www.fda.gov/regulatory-information/search-fda-guidance-documents/guidance-industry-estimating-dietary-intake-substances-food.

[81] Tristan Asensi M., Napoletano A., Sofi F., Dinu M. (2023). Low-Grade Inflammation and Ultra-Processed Foods Consumption: A Review. Nutrients.

[82] Hall K.D., Ayuketah A., Brychta R., Cai H., Cassimatis T., Chen K.Y., Chung S.T., Costa E., Courville A., Darcey V. (2020). Ultra-Processed Diets Cause Excess Calorie Intake and Weight Gain: An Inpatient Randomized Controlled Trial of Ad Libitum Food Intake. Cell Metab..

[83] Ye C., Li Z., Ye C., Yuan L., Wu K., Zhu C. (2024). Association between Gut Microbiota and Biological Aging: A Two-Sample Mendelian Randomization Study. Microorganisms.

[84] Ruiz P.A., Morón B., Becker H.M., Lang S., Atrott K., Spalinger M.R., Scharl M., Wojtal K.A., Fischbeck-Terhalle A., Frey-Wagner I. (2017). Titanium dioxide nanoparticles exacerbate DSS-induced colitis: Role of the NLRP3 inflammasome. Gut.

[85] Vallianou N.G., Evangelopoulos A., Tzivaki I., Daskalopoulou S., Adamou A., Michalaki Zafeiri G.C., Karampela I., Dalamaga M., Kounatidis D. (2025). Ultra-Processed Foods and Type 2 Diabetes Mellitus: What Is the Evidence So Far?. Biomolecules.

[86] Dunford E.K., Miles D.R., Popkin B. (2023). Food Additives in Ultra-Processed Packaged Foods: An Examination of US Household Grocery Store Purchases. J. Acad. Nutr. Diet..

[87] López-Otín C., Blasco M.A., Partridge L., Serrano M., Kroemer G. (2023). Hallmarks of aging: An expanding universe. Cell.

[88] Scrinis G., Popkin B.M., Corvalan C., Duran A.C., Nestle M., Lawrence M., Baker P., Monteiro C.A., Millett C., Moubarac J.C. (2025). Policies to halt and reverse the rise in ultra-processed food production, marketing, and consumption. Lancet.

[89] Pineda E., Poelman M.P., Aaspõllu A., Bica M., Bouzas C., Carrano E., De Miguel-Etayo P., Djojosoeparto S., Blenkuš M.G., Graca P. (2022). Policy implementation and priorities to create healthy food environments using the Healthy Food Environment Policy Index (Food-EPI): A pooled level analysis across eleven European countries. Lancet Reg. Health Eur..

[90] Lichtenstein A.H., Appel L.J., Vadiveloo M., Hu F.B., Kris-Etherton P.M., Rebholz C.M., Sacks F.M., Thorndike A.N., Van Horn L., Wylie-Rosett J. (2021). 2021 Dietary Guidance to Improve Cardiovascular Health: A Scientific Statement From the American Heart Association. Circulation.

[91] Marassi M., Fadini G.P. (2023). The cardio-renal-metabolic connection: A review of the evidence. Cardiovasc. Diabetol..

[92] Calvo M.S., Dunford E.K., Uribarri J. (2023). Industrial Use of Phosphate Food Additives: A Mechanism Linking Ultra-Processed Food Intake to Cardiorenal Disease Risk?. Nutrients.

[93] Soliman A.R., Abdelghany R., Abdelaziz T.S., Attia A., Ahmed R.M. (2025). Counting the invisible: Dietary inorganic phosphorus intake across different chronic kidney disease stages in elderly patients-a national insight. J. Health Popul. Nutr..

[94] Bernard L., Chen J., Kim H., Wong K.E., Steffen L.M., Yu B., Boerwinkle E., Levey A.S., Grams M.E., Rhee E.P. (2024). Serum Metabolomic Markers of Protein-Rich Foods and Incident CKD: Results From the Atherosclerosis Risk in Communities Study. Kidney Med..

[95] Shateri Z., Eskandarzadeh S., Nouri M., Jahromi S.E., Mansouri F., Babajafari S. (2024). The role of ultra-processed food consumption in protein-energy wasting and sarcopenia development in patients with chronic kidney diseases. BMC Nephrol..

